# Evaluation of the internal construct validity of the Personal Care Participation Assessment and Resource Tool (PC-PART) using Rasch analysis

**DOI:** 10.1186/s12913-014-0543-z

**Published:** 2014-11-05

**Authors:** Susan Darzins, Christine Imms, Marilyn Di Stefano, Nicholas F Taylor, Julie F Pallant

**Affiliations:** School of Allied Health, Australian Catholic University, Locked Bag 4115, Fitzroy, 3065 Australia; School of Allied Health, La Trobe University, Bundoora, Victoria Australia; Eastern Health, Box Hill, Victoria Australia; Rural Health Academic Centre, University of Melbourne, Shepparton, Victoria Australia

**Keywords:** Rehabilitation, Activities of daily living, Outcome assessment, Validation studies, Construct validity, World Health Organisation, Participation

## Abstract

**Background:**

The Personal Care Participation Assessment and Resource Tool (PC-PART) is a 43-item, clinician-administered assessment, designed to identify patients’ unmet needs (participation restrictions) in activities of daily living (ADL) required for community life. This information is important for identifying problems that need addressing to enable, for example, discharge from inpatient settings to community living. The objective of this study was to evaluate internal construct validity of the PC-PART using Rasch methods.

**Methods:**

Fit to the Rasch model was evaluated for 41 PC-PART items, assessing threshold ordering, overall model fit, individual item fit, person fit, internal consistency, Differential Item Functioning (DIF), targeting of items and dimensionality. Data used in this research were taken from admission data from a randomised controlled trial conducted at two publically funded inpatient rehabilitation units in Melbourne, Australia, with 996 participants (63% women; mean age 74 years) and with various impairment types.

**Results:**

PC-PART items assessed as one scale, and original PC-PART domains evaluated as separate scales, demonstrated poor fit to the Rasch model. Adequate fit to the Rasch model was achieved in two newly formed PC-PART scales: *Self-Care* (16 items) and *Domestic Life* (14 items). Both scales were unidimensional, had acceptable internal consistency (PSI =0.85, 0.76, respectively) and well-targeted items.

**Conclusions:**

Rasch analysis did not support conventional summation of all PC-PART item scores to create a total score. However, internal construct validity of the newly formed PC-PART scales, *Self-Care* and *Domestic Life*, was supported. Their Rasch-derived scores provided interval-level measurement enabling summation of scores to form a total score on each scale. These scales may assist clinicians, managers and researchers in rehabilitation settings to assess and measure changes in ADL participation restrictions relevant to community living.

**Trial registration:**

Data used in this research were gathered during a registered randomised controlled trial: Australian and New Zealand Clinical Trials Registry ACTRN12609000973213. Ethics committee approval was gained for secondary analysis of data for this study.

## Background

Participation is described in the International Classification of Functioning, Disability and Health (ICF) as a person’s involvement in a life situation. Participation restrictions are described as problems a person may have in their involvement in a life situation [1]. Activities are described as execution of tasks or actions by a person. Activity limitations are difficulties a person may have in executing activities [1]. Much has been said about the ICF’s lack of clarity in these definitions and the difficulties operationalizing these concepts [2-5]. To date, there is no consensus about the definition of the concept of participation (restriction), nor the measurement of participation (restriction) [4].

There is division amongst researchers as to whether Self Care and Domestic Life tasks classified within the ICF belong to the activity or to the participation construct [2,6]. Such allocations have generally been made according to content of the categories within these domains. The distinction between measurement of constructs that are more closely aligned to activity (limitation) versus participation (restriction) may depend not only on the content of the items within an instrument, but also on the metric used in the measure [3]. Measures eliciting information about an individual’s ability, level of difficulty, level of dependence in performing tasks, without inclusion of the modifying effects of the environment in the metric, are more closely aligned to the measurement of activity (limitation). Measures eliciting information about actual performance of, and satisfaction with, tasks in environments where they occur, and which include in the metric, influences of the environment and health condition on performance and satisfaction, are more closely aligned to the measurement of participation (restriction) [3].

The Personal Care Participation Assessment and Resource Tool (PC-PART) records the transaction between the person, their health condition and environmental factors operating in the person’s living situation, resulting in measurement of the person’s met and unmet ADL needs in their living situation (life situation). It is important to measure both met and unmet ADL needs in order to understand the nature and extent of problems people experience accomplishing both self care and domestic life activities of daily living required for community life, and their involvement in community living as a citizen. Unmet ADL needs, as measured by the PC-PART, are aligned to the construct of participation restriction and are therefore named ADL participation restrictions.

The PC-PART is divided into 43 items across seven domains: *Clothing*; *Hygiene*; *Nutrition*; *Mobility*; *Safety*; *Residence*; and *Supports*. It is a clinician-administered assessment and uses a structured interview format to gather information and item responses from the person and if necessary, key informant(s). Item responses are: *OK by self* (patient manages activity alone with or without aids and appliances in the living environment), *OK with help* (patient manages activity with help from others, and this help is available in the living environment), or *Not OK* (patient does not manage the activity in the living environment despite their own efforts, use of aids and appliances and help from available support from others). Both *OK by self* and *OK with help* are scored 0, and *Not OK* is scored 1, forming a dichotomy. Each *Not OK* represents an ADL participation restriction and provides a target for intervention. The final domain, *supports*, consists of two questions addressing the adequacy and stability of available supports, with responses *OK* and *Not OK.* Conventional overall scoring of the PC-PART involves summation of *Not OK* responses to produce a total score, producing ordinal data.

There is an important and clinically relevant distinction between the PC-PART and other ADL measures. Commonly used ADL instruments in Australia, such as the FIM [7] and Barthel Index (BI) [8] measure a person’s capabilities and their individual level of independence/dependence in self care activities of daily living and mobility. These are therefore measures of activity (limitations). While this is clinically important information to gather, such ADL measures stop short of measuring actual accomplishment of activities of daily living in the person’s living environment. This is because they do not incorporate into the measurement, the availability and stability of specifically needed assistance to the person in their living environment. This latter information is clinically relevant. For example, for discharge planning, it is the aim of health services to address people’s ADL needs required for community living before returning people to live in the community. The PC-PART was designed to achieve this.

There are other ADL measures that specifically address supports, resources or assistance (environmental barriers and facilitators) as part of their responses and scoring, including the Assessment of Living Skills and Resources-Revised 2 (ALSAR-R2) [9], Assessment of Life Habits (LIFE-H) [10], Craig Handicap Assessment and Reporting Technique (CHART) [11] and the Functional Autonomy Measurement System (SMAF) [12]. However, the ALSAR-R2, LIFE-H and CHART cover broader areas of functioning than the PC-PART (such as education, work and leisure) and therefore have application in different environments from the PC-PART. The SMAF was developed for the measurement of care needs in older adults in order to allocate community services or chronic care beds [12]. It was not developed for use with younger people. While the SMAF covers essential self care and domestic life activities of daily living, it differs from the PC-PART in that it also includes items focused on body functions (e.g. vision, speaking, hearing, memory). Each instrument described above varies in the way it incorporates the availability and the need for supports, resources or assistance into the instrument’s scoring. The PC-PART is the only instrument we are aware of that specifically targets the transaction between the person, the activity and the available supports in the person’s living environment to record participation restrictions in those activities of daily living required for community life.

The PC-PART has demonstrated content validity [13] and good inter-rater reliability for grouped data [14-16], but its internal validity has not been subjected to rigorous evaluation [15]. Whether it is valid to sum PC-PART item scores has not been tested. For clinicians, health-service managers and researchers to have confidence in any measurement instrument, and the scores derived from it, evidence of internal and external validity of the instrument is required. Therefore, the aim of the current study was to evaluate the internal construct validity of the PC-PART to address this gap in the tool’s validation, and to refine the instrument, if necessary, using Rasch methods [17].

## Methods

### Design

This was an instrument validation study. Methods based in Item Response Theory have increasingly been used to evaluate psychometric properties of health measures, and have been applied to both personal and instrumental ADL instruments [18-22]. The Rasch model is a one-parameter model within the Item Response Theory framework [23,24]. It involves formal rigorous psychometric testing of a scale against a mathematical measurement model by testing the scale’s fit to the Rasch model [17,25,26]. The model asserts that scale item scores can only be appropriately summed to provide a total score if the scale is unidimensional. If items satisfy expectations of the Rasch model, the analysis enables transformation of the scale’s ordinal raw scores to interval-level measurement [26,27]. Methods based in Classical Test Theory (CTT), such as Factor Analysis and Confirmatory Factor Analysis, were not appropriate for this study because PC-PART items violate assumptions that scale items have interval-level properties [24].

### Participants

This study involved secondary analysis of data from 996 adult inpatient rehabilitation participants in Melbourne, Australia, enrolled in a trial of standard versus augmented therapy (ACTRN12609000973213) [28]. The PC-PART was administered as an outcome measure at admission to, and at discharge from the inpatient rehabilitation unit. Participants were included in the trial if they were aged 18 years or older, were admitted for rehabilitation to one of two government-funded hospital facilities and consented to participate in the trial. Patients were excluded if they were admitted for geriatric evaluation and management, or if they were already enrolled in another intervention trial. The rehabilitation setting provided therapeutic intervention and multi-disciplinary management.

Participants’ admission PC-PART data were used in this study. The PC-PART was administered by an occupational therapist blinded to group allocation. The occupational therapist completed PC-PART assessments using a combination of patient interview, key informant interview and task observation. The occupational therapist assessor was provided with standardized education in the use of the PC-PART prior to commencement of data collection. This consisted of a one-hour training session with an occupational therapist experienced in use of the PC-PART. In addition, the PC-PART manual [29] and DVD [30] were made available.

This secondary analysis of trial data was approved by Human Research and Ethics Committees at Eastern Health (E58/0910) and La Trobe University (FHEC10/14).

### Data analysis

Rasch modelling procedures consistent with established guidelines were adopted [25-27,31,32], using RUMM 2030 software [33]. For a 41-item scale, a sample size of 250 for well-targeted items, or 820 for poorly-targeted items, provides 99% confidence that person estimates are definitive [34]. Therefore, the sample of 996 in the current study was adequate.

Analysis methods and criteria applied to tests of fit to the Rasch model included assessment of (1) overall fit to the Rasch model; (2) item response format; (3) individual item fit; (4) individual person fit; (5) Differential Item Functioning (DIF); (6) internal consistency; (7) local dependency among items; (8) dimensionality of the scale, and (9) targeting of items.

In large samples and with scales involving large numbers of items, the chi-square statistic may not be a reliable indicator of fit to the Rasch model. Therefore, in this study, other fit statistics were used. Overall fit to the model was observed using Fit Residual values, with a Fit Residual Standard Deviation value exceeding 1.5 suggesting possible misfit. To assess fit of individual items and persons to the scale, it was expected that the individual item and person Fit Residual values should fall within the range of +/− 2.5 [27].

Problems with an item’s response format were indicated by the presence of disordered thresholds. A threshold is the point between two response categories where either response is equally probable. Inconsistent use of item response categories results in disordered thresholds. Presence of disordered thresholds indicated the need to reduce the number of response categories [25,27].

Differential Item Functioning (DIF) occurs when different groups within the same sample (e.g. men and women) respond differently to an item despite having equal levels of the underlying trait. Both uniform (systematic) and non-uniform (not systematic) DIF by age and sex were examined. Items displaying DIF were evaluated for their clinical importance to the scale versus the potential for improvement of the internal validity of the scale resulting from their removal [27]. The Person Separation Index (PSI) provided an indication of the internal consistency of the scale and the power of the scale to discriminate amongst persons with different levels of the trait. A value of at least 0.7 was considered acceptable [25].

Local dependency between item-pairs was considered to exist when the response to one item was dependent on the response to another item, revealing between-item residual correlations matrix values above 0.2. Item-pairs showing local dependency above 0.2 were examined for potential item-redundancy using clinical judgement. Items were further examined to identify if retaining both items inflated the scale’s PSI value. This was assessed by forming ‘subtests’, joining locally dependent item pairs, to absorb the effect of the dependent items on PSI [25]. If the PSI value then changed by more than +/− 0.1, consideration was then given to removal of one of the locally dependent items from the scale.

To test dimensionality of the scales, items with strongest positive and negative loadings from the first component of the Principal Components Analysis of the standardised residuals were used in a series of independent t-tests to test the null hypothesis of no difference in the individual person location scores between the two sets of items. If fewer than 5% of the t-tests showed statistically significant differences, or the lower bound value of the associated 95% confidence interval was 5% or lower, then the scale was considered unidimensional [26,31,35].

Targeting of items in the scale was checked with a person-item map to evaluate if there were sufficient items to measure the full extent of clinically relevant ADL participation restrictions among persons, without ceiling effects [25,27]. Floor effects were not considered relevant in this evaluation, as clinical teams are more concerned about addressing the presence of ADL participation restrictions, rather than the absence of participation restrictions prior to discharge from the hospital setting.

Rasch analysis was conducted in three stages on 41 PC-PART items listed in Table 1, column 1. The two *Supports* items were excluded from all analyses as they were considered to be global items, measuring a different construct to the remaining PC-PART items. During Stage one of the analysis, the 41 items were analysed as one scale, consistent with the recommended scoring protocol. The alternative three-category item response options (0 = *OK by self*, 1 = *OK with help* and 2 = *Not OK*) were also evaluated to determine if they were appropriate for use, instead of the existing two-category item response options (0 = *OK by self*, 0 = *OK with help* and 1 = *Not OK)*. In Stage two of the analysis, fit to the Rasch model was evaluated for the six original PC-PART domains (*Clothing*, *Hygiene*, *Nutrition*, *Mobility*, *Safety* and *Residence)* using the two and three-category response options just described*.*

**Table 1:** **Original PC-PART domains and items and refined PC-PART** ***Self Care*** **and** ***Domestic Life*** **scales**

**1. Original PC-PART** **domains and items**	**2. Items included in the Rasch-derived scales**		**3. Items not included in the Rasch-derived scales.**
	***(a) Self Care***	**(b)** ***Domestic Life***	
	**16 items**	**14 items**	
**A. Clothing**			
A1 Manage dressing: top (upper body)	✓		
A2 Manage dressing: bottom (lower body)	✓		
A3 Getting socks & shoes on/off	✓		
A4 Select clothing appropriate for weather	✓		
A5 Managing laundry		✓	
**B. Hygiene**			
B1 Manage toileting	✓		
B2 Bladder control/keeping pants dry			✓
B3 Bowel control/keeping pants unsoiled			✓
B4 Washing hair			✓
B5 Cleaning teeth	✓		
B6 Manage shaving/menstruation			✓
B7 Washing self	✓		
B8 Getting in & out of bath/shower	✓		
**C. Nutrition**			
C1 Maintaining usual weight			✓
C2 Eat without choking/coughing			✓
C3 Planning meals		✓	
C4 Preparing meals		✓	
C5 Acquiring groceries		✓	
C6 Managing food restrictions	✓		
C7 Using the stove		✓	
C8 Avoiding spoiled food	✓		
**D. Mobility**			
D1 Moving around inside the home	✓		
D2 Getting in & out of bed	✓		
D3 Move around without falling	✓		
D4 Managing steps/stairs	✓		
D5 Moving around outdoors		✓	
D6 Driving safely			✓
D7 Getting to/from appointments		✓	
D8 Wandering (remember where to go without getting lost)			✓
D9 Orientation (remembering appointments)			✓
**E. Safety**			
E1 Managing medications		✓	
E2 Avoiding alcohol/substance overuse		✓	
E3 Coping with minor illness/crisis	✓		
E4 Coping without repeated emergency help		✓	
E5 Managing safety hazards when smoking			✓
E6 Home free of hazards			✓
**F. Residence**			
F1 Managing money		✓	
F2 Managing home security		✓	
F3 Using basic personal Information		✓	
F4 Shopping for personal/household needs		✓	
F5 Keep cool in Summer /warm in Winter	✓		
**G. Supports**			
G1 Adequacy of supports from others			✓
G2 Stability of supports from others			✓

NOTE: G1 and G2 were not included in the Rasch Analysis.

Stage three of the analysis involved forming alternative PC-PART item groupings using the ICF as the theoretical framework a-priori to further analysis. PC-PART items were linked to ICF categories using Cieza’s linking rules [36,37]. Most items aligned to either the *Self-Care* or *Domestic Life* chapter of the ICF *activities and participation* component [1]. Items that aligned to other ICF chapters, such as *mobility*, were assigned to either the *Self-Care* or *Domestic Life* item group based on the activity context of the mobility item. *Self-Care* items corresponded to personal ADL activities, for example, bathing, toileting, dressing and eating. *Domestic Life* items corresponded to broader instrumental ADL activities needed for community living, for example, shopping, transportation, laundry and food preparation. The newly formed *Self-Care* and *Domestic Life* item groups were then evaluated for their fit to the Rasch model.

## Results

### Participants

Participants’ mean (SD) age was 73.9 (12.8) years, with a minimum of 22 years and a maximum of 102 years and 631 (63%) were women. A total of 581 (58%) participants were admitted with an orthopaedic impairment, 203 (20%) with neurological impairment and 212 (21%) with other disabling impairments. Prior to admission, 94% of participants had been living in their own homes, while 3% lived in ‘low-level’ residential care facilities. These admission data are typical of Australian inpatient rehabilitation settings [38]. Complete admission PC-PART data were available for 958 (96%) of the 996 participants.

Table 2 displays results from the three-staged analysis.

**Table 2:** **Model fit for the three-staged Rasch analysis (n = 958 in each analysis)**

**Subscale**	**No. of items**	**Item response categories**	**Overall model fit** ^**a**^	**Overall Item fit residual (SD)** ^**b**^	**Overall Person fit residual (SD)** ^**b**^	**No. of items with disordered thresholds**	**No of misfitting items** ^**c**^	**No of misfitting persons** ^**c**^	**PSI** ^**d**^	**Items with uniform/non-uniform DIF**	**No. of item-pairs with response dependency** ^**e**^	**Dimensionality**
										**(a) by age**		**(% of significant t-tests)** ^**f**^
										**(b) by sex**		
**Stage 1. All PC-PART items as one scale**												
(a) All original items	41	012	χ^2^ = 2003.85, df (369), *p* < .001, (α = .001)	−0.07 (2.84)	−0.46 (1.23)	27	9 (D6 C1 E6 C5 E1 F4 E2 D7 C7)	21	0.93	(a) B2 D6 E2/ –	48	11.5%
										(b) A5 B6 B7 C3 D9/ –		(95% CI 10.1-12.9%)
(b) All original items	41	001	χ^2^ = 1743.19, df (369), *p* < .001, (α = .001)	−0.47 (2.14)	−0.54 (1.03)	NA^g^	3 (D6 D7 C1)	11	0.91	(a) B2 D6 E2/ –	39	7.1%
										(b) A5 B6 B7 C4/ E3		(95% CI 5.7-8.5%)
(c) Removal of 6 items	35	001	χ^2^ = 693.50, df (234), *p* < .001, (α = .001)	−0.46 (1.80)	−0.52 (0.87)	NA	0	6	0.88	(a) B2 E2/ –	5	7.52%
										(b) C4/ –		(95% CI 6.1-8.9%)
**Stage 2. Original PC-PART domains**												
Clothing	5	001	χ^2^ = 316.31, df (15), *p* < .001, (α = .01)	−0.81, (4.23)	−0.34, (0.60)	NA	1 (A5)	0	0.54	(a) –/ –	1	–^h^
										(b) A5/ –		
Hygiene	8	001	χ^2^ = 384.92, df (39), *p* < .001, (α = .006)	−1.03 (1.99)	−0.75 (0.86)	NA	0	0	0.68	(a) B2/ –	1	1.98%
										(b) –/ B2 B6		
Mobility	9	001	χ^2^ = 539.17, df (54), *p* < .001, (α = .006)	−1.51 (3.38)	−0.42 (0.48)	NA	2 (D6 D7)	0	0.68	(a) D3 D6/ –	2	1.67%
										(b) –/ –		
Nutrition	8	001	χ^2^ = 413.10, df (48), *p* < .001, (α = .006)	−1.04 (3.05)	−0.53 (0.71)	NA	2 (C1 C5)	0	0.49	(a) –/ C6	0	4.92%
										(b) C3/ –		
Residence	5	001	χ^2^ = 189.26, df (5), *p* < .001, (α = .01)	−3.18 (2.9)	−0.59 (0.50)	NA	0	0	−0.31	(a) –/ –	0	–^h^
										(b) –/ F3		
Safety	6	001	χ^2^ = 109.40, df (23), *p* < .001, (α = .008)	−0.44 (2.62)	−0.25 (0.48)	NA	0	0	−0.46	(a) E2/ –	0	0.00%
										(b) –/ –		
**Stage 3. PC-PART items separated into ‘Self Care’ and ‘Domestic Life’ scales**												
(a) All self care items	23	001	χ^2^ = 987.96, df (184), *p* < .001, (α = .002)	−0.64 (2.33)	−0.51 (0.77)	NA	3 (C1 C2 B2)	2	0.87	(a) B2/ –	11	14.61%
										(b) B6/ –		(95% CI 13.2%-16.0%)
All domestic life items	18	001	χ^2^ = 1058.02, df (162), *p* < .001, (α = .002)	−0.77 (2.48)	−0.50 (0.81)	NA	2 (D6 E6)	0	0.79	(a) E2/ –	7	8.56%
										(b) A5 C4/ –		(95% CI 7.2%-9.9%)
(b) Refined Self Care scale	16	001	χ^2^ = 360.64, df (91), p < .001, (α = .003)	−0.86 (1.87)	−0.46 (0.62)	NA	0	0	0.85	(a) –/ –	0	4.18%
										(b) –/ –		
Refined domestic life scale	14	001	χ^2^ = 515.48, df (91), p < .001, (α = .004)	−0.57 (2.02)	−0.45 (0.63)	NA	0	0	0.76	(a) E2/ –	0	6.16%
										(b) A5 C4/ –		(95% CI 4.8-7.5%)

SD = Standard Deviation, PSI = Person Separation Index, DIF = Differential Item Functioning, χ^2^ = chi square, df = degrees of freedom, *p* = probability value, α = Bonferroni adjusted alpha level, CI = Confidence Interval.

^a^non significant chi-square item-trait interaction statistic is evidence of overall model fit.

^b^Item-Person Fit Residual SD ≤1.5 is evidence of overall item/person fit.

^c^Individual item or person Fit Residual values of ≤2.5 are evidence of item/person fit.

^d^PSI values ≥0.7 acceptable for use with grouped data, values ≥0.8 acceptable for use with individual data.

^e^Item pairs with Residual correlation values of r ≥0.2 deemed to have local item dependency.

^f^Values ≤5% considered evidence of a unidimensional scale (95% CI only presented when the proportion of significant t-tests exceeded 5%).

^g^Not Applicable - only one threshold for items with two response categories.

^h^Insufficient items and response categories to produce minimum scores for meaningful results.

### Stage 1. One scale containing 41 PC-PART items

During stage 1(a) of the analysis, when assessed using the three response categories (0,1,2), 27 of the 41 PC-PART items showed disordered thresholds, suggesting the need to collapse the response categories to form a dichotomous scale (0,0,1).

In stage 1(b) of the analysis using the dichotomous scale, there was evidence of overall item misfit, with the overall item fit residual standard deviation (SD) being 2.14 (≥1.5), and the presence of three misfitting items. There were 11 misfitting persons. Internal consistency of the scale was high (PSI = 0.91). There was evidence of uniform DIF by age (three items) and sex (four items) and non-uniform DIF by sex (one item). Local item dependency was observed for 39 item-pairs. The scale was not unidimensional, with the lower bound 95% CI of the proportion of significant t-tests (5.7%) being above the critical value of 5%.

Attempts were made to refine the scale to achieve unidimensionality and fit of the scale to the Rasch model in stage 1(c) of the analysis. With removal of six misfitting items, the overall item fit residual standard deviation (SD) was reduced to 1.8. While there were no misfitting items and PSI was acceptable (0.88), there was evidence of uniform DIF by age (two items) and sex (one item) and there were five item-pairs with local dependency. Additionally, the scale was not unidimensional, with the lower bound 95%CI value on the proportion of significant t-tests being 6.1%. A decision was made to move to Stage 2 of the analysis.

### Stage 2. Original PC-PART domains

Rasch analysis of six original PC-PART domains using the three response categories (0,1,2) revealed disordered thresholds for all six domains. Therefore, the response categories were collapsed to the original dichotomous responses (0,0,1) and the Rasch analysis was repeated. While four domains had sufficient items to test dimensionality and appeared to be unidimensional, overall fit to the Rasch model was poor. All six domains showed inflated item fit residual SDs (range 1.99 to 4.23). Item misfit was detected in three of the six domains. PSI values in all domains were below the critical value of 0.7. Uniform DIF by age was present for *Hygiene* (one item), *Mobility* (two items), and *Safety* (one item), and by sex for *Clothing* (one item) and *Nutrition* (one item). Non-uniform DIF by age was present for *Nutrition* (one item) and by sex for *Hygiene* (two items) and *Residence* (one item). There was local item response dependency for *Clothing (one item-pair), Hygiene (one item-pair) and Mobility (two item-pairs)*. Fit to the Rasch model deteriorated further through attempts to refine the original domain scales by deleting misfitting items. Therefore the decision was made to move to Stage 3 of the analysis.

### Stage 3. PC-PART items separated into ‘Self-Care’ and ‘Domestic Life’ scales

*Stage 3(a).* Rasch analysis was conducted on the proposed *Self-Care* (23 items) and *Domestic Life* (18 items) scales using the dichotomous item response categories (0,0,1). The 23 *Self-Care* items showed evidence of misfit (Item Fit Resid. SD =2.33), with three misfitting items and two misfitting persons. The PSI was acceptable (PSI = 0.87). Only uniform DIF was present for one item by age and one item by sex. Local item response dependency was present for 11 item pairs. The scale failed the test for unidimensionality. Analysis of the 18 *Domestic Life* items revealed overall misfit (Item Fit Resid. SD =2.48), with two misfitting items and no misfitting persons. PSI was acceptable (PSI = 0.79). Uniform DIF was present for one item by age and two items by sex. There was evidence of local item response dependency for seven item-pairs. The scale failed the test for unidimensionality.

*Stage 3(b).* Refinement of the *Self-Care* scale involved deletion of seven misfitting or redundant items. Although the resultant *Self-Care* scale containing 16 items showed slightly elevated overall item fit residual statistics (Item Fit Resid. SD =1.87), there was no individual item misfit and no misfitting persons. The PSI (0.85) was acceptable. There was no evidence of DIF by age or sex. There was no local item response dependency and the scale was shown to be unidimensional. The 16 *Self-Care* scale items in the refined scale are shown in Table 1, column 2a. Refinement of the *Domestic Life* scale involved deletion of four items and creation of one subtest between items showing local dependency. The refined scale, containing 14 items, had no misfitting items or persons. The PSI (0.76) was acceptable. There was uniform DIF by sex for items ‘laundry’ and ‘meal preparation’, with women scoring higher than men; and by age for the item ‘avoiding alcohol/substance abuse’, with younger patients showing higher scores than older patients. There was no local item dependency. The scale was shown to be unidimensional with the lower bound 95%CI of the percentage of significant t-tests being 4.8%. The 14 *Domestic Life* scale items on the refined scale are shown in Table 1, column 2b.

**Figure 1:**
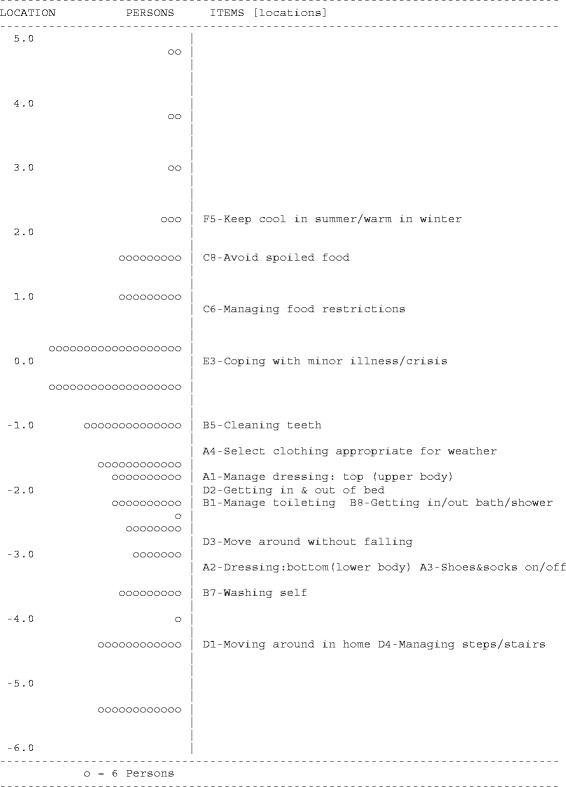
**Item map for the PC-PART** ***Self Care*** **scale.** Location values for persons are on the left (o =6 Persons). Relative difficulty of items is displayed on the right. Items at higher location scores represent activities that are Not OK for relatively few people; only people with higher levels of ADL participation restriction are rated ‘Not OK’ on these items. These are ‘easier’ items for most people to manage. Items at lower location scores represent activities that are Not OK for relatively many people; people with lower levels of ADL participation restriction are rated NOT OK on these items. These are ‘harder’ items for most people to manage.

**Figure 2:**
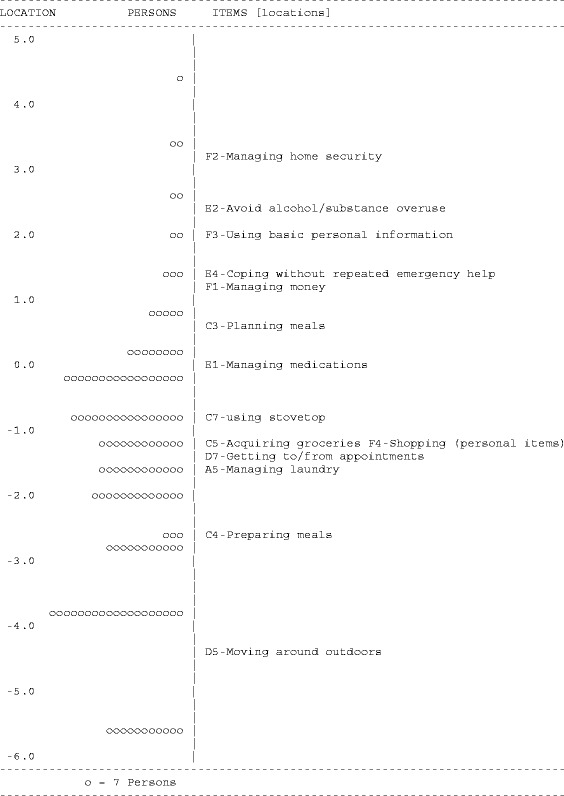
**Item map for the PC-PART** ***Domestic Life*** **scale.** Location values for persons are on the left (o =7 Persons). Relative difficulty of items are displayed on the right. Items at higher location scores represent activities that are Not OK for relatively few people; only people with higher levels of ADL participation restriction are rated Not OK on these items. These are ‘easier’ items for most people to manage. Items at lower location scores represent activities that are Not OK for relatively many people; people with lower levels of ADL participation restriction are rated NOT OK on these items. These are ‘harder’ items for most people to manage.

Item-location maps for the refined *Self-Care* and *Domestic Life* scales (Figures 1 and 2) suggested items were well targeted, demonstrating sufficient item spread across the full range of person location scores on both scales, without ceiling effects. Higher scores on the *Self-Care* and *Domestic Life* scales indicated higher (worse) levels of *Self-Care* and *Domestic Life* ADL participation restriction.

#### Combined self-care and domestic life scales

Dimensionality testing was completed including all 30 items from the resultant *Self-Care* and *Domestic Life* scales in one analysis. This scale failed the test for unidimensionality, with the 95% CI for the percentage of significant t-tests ranging from 5.8% to 8.6%. Therefore summation of *Self-Care* and *Domestic Life* scale scores to form a total PC-PART score was not supported.

#### Conversion scores

Adjusted conversion scores were computed from the Rasch-derived logit scores on the refined *Self-Care* and *Domestic Life* scales, using a 0 to 100 scale, with higher scores indicating higher levels of participation restriction. This enabled conversion of raw ordinal scores from the scales to interval level measurement. For practical purposes, a converted score is dependent on all items in the scales being answered. The mean *Self-Care* admission converted score was 42.0 (N = 958; SD = 22.3; Range 0,100) and the mean *Domestic Life* admission converted score was 38.5 (N = 957; SD =20.4; Range 0,100). These scores represented between 6/7 and 4/5 ADL participation restrictions (raw scores) on the scales, respectively.

## Discussion

Rigorous psychometric analysis was used to examine the internal construct validity of the PC-PART in order to enhance empirical development of the tool [15]. Rasch analysis demonstrated that it is inappropriate to sum all items in the original PC-PART item set to produce a total score, and that the six original PC-PART domains did not form psychometrically sound scales. Use of Rasch methods generated evidence supporting the internal construct validity of the newly formed PC-PART *Self-Care* (16 items) and *Domestic Life* (14 items) scales as measures of *Self Care* and *Domestic Life* ADL participation restriction. These were shown to be unidimensional scales. The total raw scores on each scale may be matched to corresponding Rasch-derived conversion scores on a 0 to 100 scale, for use as interval-level measurement (conversion scores available from the corresponding author).

Frequently used and researched self care and domestic life ADL measures [7,8,39] typically measure patients’ level of dependence (i.e. activity limitations). One shortcoming of this approach is that decisions about whether patients are ready for discharge from inpatient settings depends not only on what patients can or cannot do for themselves, but how they will complete self care and domestic life ADL in their real living environment with the supports that are available; in other words whether or not there will be unmet self-care and domestic life ADL needs (participation restrictions) [40,41]. The PC-PART *Self-Care* and *Domestic Life* scales address this limitation through the measurement of ADL participation restrictions. These scales may be used alongside existing measures of ADL in/dependence, to enable more complete and useful measurement of patients’ ADL functioning for community life. Such measurement of ADL functioning may enable existing barriers to patients’ discharge to community living to be identified and addressed [41,42]. In this way, the PC-PART scales may assist decision-making by health care team, consistent with the original purpose of the PC-PART [13,29].

The PC-PART *Self-Care* and *Domestic Life* scales may have potential to aid health care system management. The patterns and the extent of ADL participation restrictions experienced by specific patient populations, as well as the extent of care required by family, friends and neighbours in providing support to those who need it, is an inadequately described phenomenon [43,44]. The PC-PART scales may enable identification and documentation of unmet ADL needs that arise from inadequate and/or unstable supply of both formal and informal supports intended to enable people to accomplish essential self-care and domestic life activities in their community living environments. Support with self-care activities (e.g. toileting, showering, and dressing) and domestic life activities (e.g. shopping, cooking, transport, and household tasks) is commonly provided by a combination of both formal and informal supports including family, neighbours, friends and paid or volunteer services [45]. Use of the PC-PART scales may assist clinicians, managers and researchers to quantify the extent of informal supports that help people accomplish their essential activities of daily living. The involvement of patients and their key informants in the PC-PART assessment may enable identification of the types of supports and resources most needed in communities by specific patient groups, as well as identification of existing service gaps. Recent literature highlights the importance of involving patients and carers in identifying the types of supports that would be of greatest assistance to them in easing carer strain [43-46].

The PC-PART scales provide interval level measurement, which may be used to measure the magnitude of change in patients’ levels of ADL participation restriction. This may make it possible to investigate the efficiency of clinical interventions and community services that seek to reduce ADL participation restrictions. This may be of significance for outcome-based payment systems. In Australia, the most recent payment system incorporates measurement of functioning across a limited number of domains, focusing on measuring activity limitations, and this may not be adequate for complex rehabilitation [41]. Madden et al. reported there is a need for an ICF-linked standardised measure within case-mix systems, and that including information about broad aspects of functioning increases the proportion of the variance explained in health care costs [41]. The PC-PART may be an appropriate measure for this purpose.

One of the strengths of this study was the use of Rasch analysis to provide a detailed analysis of not only the PC-PART items, but also the item response categories [24,25]. Analysis of the PC-PART’s item response categories supported use of the dichotomous response categories of the PC-PART items. These response categories are consistent with the overall purpose of the instrument, which is to identify and document the presence of ADL participation restrictions in activities of daily living required for community life.

The presence of uniform DIF by age in the *Domestic Life* scale for ‘avoiding alcohol/substance overuse’ and by sex for ‘managing laundry’ and ‘meal preparation’ suggested influences on scores associated with age and sex, respectively. While it is usual to delete items that demonstrate DIF, these items were retained because they were deemed to be clinically relevant to the scale and the observed DIF could be clinically explained. Further validation of the scales would provide additional evidence about the appropriateness of retaining these items.

An inter-rater reliability study of the PC-PART conducted in the same rehabilitation centres, using the same therapists to collect PC-PART data, with an independent sample of patients, showed a high level of inter-rater agreement, with an intra-class correlation coefficient of 0.91 (95% CI 0.88 to 0.93) for grouped PC-PART data [16]. Hence, it is unlikely that potential measurement error during data collection influenced the results of this present study.

Of the original PC-PART items, 13 showed misfit during the Rasch scale refinement process, and were excluded from the newly formed PC-PART *Self Care* and *Domestic Life* scales. However, it is still possible that some of these items may be clinically relevant as part of an assessment of ADL participation restrictions for community living. Some of the excluded items may not have had health consequences if left unmanaged, or they may have addressed different constructs to ADL participation restriction, or the aspect of ADL participation restriction covered by the item was already addressed by another item. Some items may have contained ambiguous phrasing resulting in misinterpretation by therapists.

Further investigation of the measurement properties of the PC-PART *Self-Care* and *Domestic Life* scales, including their convergent and divergent validity, longitudinal validity and criterion validity, would guide judgement regarding their utility. Specifically, investigation concerning possible cut-point scores on the PC-PART *Self-Care* and *Domestic Life* scales to indicate the critical value for inpatient care versus community living (including supported living), would provide clinically relevant information.

## Conclusions

This study generated evidence supporting the internal construct validity of the PC-PART *Self-Care* and *Domestic Life* scales as valid, unidimensional scales for inpatients receiving rehabilitation, allowing summation of scores on each scale. Rasch-derived conversion scores enable interval-level measurement, appropriate for parametric analyses of grouped data. The scales may be useful to clinical practice, clinical research and to health care system managers. Further validation research of the scales to confirm their utility is recommended.

## Abbreviations

ADL: Activities of Daily Living
DIF: Differential Item Functioning
ICF: International Classification of Functioning, Disability and Health
PC-PART: Personal Care-Participation Assessment and Resource Tool
PSI: Person Separation Index
RCT: Randomised Controlled Trial
